# Evidence of quantum phase transition in real-space vacuum entanglement of higher derivative scalar quantum field theories

**DOI:** 10.1038/s41598-017-15858-9

**Published:** 2017-11-17

**Authors:** S. Santhosh Kumar, S. Shankaranarayanan

**Affiliations:** 1 School of Physics, Indian Institute of Science Education and Research Thiruvananthapuram, CET Campus, Thiruvananthapuram, 695 016 Kerala India; 2 Department of Physics, Indian Institute of Technology Bombay, Mumbai, 400 076 Maharashtra India

## Abstract

In a bipartite set-up, the vacuum state of a free Bosonic scalar field is entangled in real space and satisfies the area-law— entanglement entropy scales linearly with area of the boundary between the two partitions. In this work, we show that the area law is violated in two spatial dimensional model Hamiltonian having dynamical critical exponent *z* = 3. The model physically corresponds to next-to-next-to-next nearest neighbour coupling terms on a lattice. The result reported here is the first of its kind of violation of area law in Bosonic systems in higher dimensions and signals the evidence of a quantum phase transition. We provide evidence for quantum phase transition both numerically and analytically using quantum Information tools like entanglement spectra, quantum fidelity, and gap in the energy spectra. We identify the cause for this transition due to the accumulation of large number of angular zero modes around the critical point which catalyses the change in the ground state wave function due to the next-to-next-to-next nearest neighbor coupling. Lastly, using Hubbard-Stratanovich transformation, we show that the effective Bosonic Hamiltonian can be obtained from an interacting fermionic theory and provide possible implications for condensed matter systems.

## Introduction

Quantum field theory plays a crucial role in understanding some of the interesting features of low-temperature condensed matter systems^1,2^. More importantly, it is well studied in the case of quantum phase transitions (QPTs) — transitions at absolute zero — where the abrupt change in the ground state properties of many body systems is governed by coherent quantum fluctuations resulting from the Heisenberg uncertainty principle^3–14^. The transition from one quantum phase to another is brought about due to the changes in the external parameter $(P)$ of the system described by the Hamiltonian $H(P)$. As like classical phase transition, at quantum critical point (QCP), there are long-range correlations in the system^9,12,13^ and that the state of the system is strongly entangled^11,15^. Hence, it is expected that quantum entanglement across regions may play a crucial role at QCP^7,10,16,17^.

In order to overcome the complexity of the interactions underlying QPTs, theoretical work has focused on one-dimensional quantum systems^11–13,18^. In this work, we study the effect of next-to-next-to-next nearest neighbor (NNN) coupling terms on the quantum fluctuations. More specifically, we consider a two-dimensional model that corresponds to NNN coupling terms on a lattice. Interesting features of the model are as follows: (i) It corresponds to the Hamiltonian of a system of coupled harmonic oscillator and hence one can compute all the relevant quantities analytically. (ii) The celebrated entanglement entropy-area law is valid for systems having local interactions^19–21^. We show violation of area law due to the presence of NNN coupling terms on a lattice. (iii) The dynamical critical exponent — a quantity that measures the scaling anisotropy between space-time variables — for our model is *z* = 3. This needs to be contrasted with the quantum Lifshitz transitions^2,22,23^ where the dynamical critical exponent is *z* = 2 and QCP is conformally critical^24,25^.

Although there is yet no fundamental understanding on the role of quantum correlations, there is a huge body of work to identify quantum information theoretic tools indicating quantum criticality^26,27^: quantum fidelity drastically changes due to the external parameter (*P*) thus signifying the notion of quantum order parameter^28^. ${\psi }_{{\rm{G}}S}(P)\in  {\mathcal H}$ (Hilbert space), the associated reduced density matrix of its subsystem can be written as ${\rho }_{{\rm{r}}ed}=exp(-{h}_{{\rm{E}}})$ ^29^, where *h*_E_ is the entanglement Hamiltonian of any one of the subsystem. It has been shown that entanglement spectrum contains more information than the entanglement entropy^30,31^. Entanglement Hamiltonian is well studied in the case of fractional quantum hall states^30^, one dimensional quantum spin systems^32,33^. In particular, at QCP, there is a finite ‘energy gap’ of *h*_E_^32,34,35^. This gap is generally termed as the entanglement gap or Schmidt gap which is considered as an order parameter to diagnose QPTs^32,33^. More importantly, the fingerprints of the topological order is encoded in the entanglement spectra^30,36–38^.

The rest of the article is organised in the following manner: In the section titled *Model and Setup*, we discuss the model Hamiltonian and the method for studying the entanglement properties. The section also contains details about mapping the model Hamiltonian to a system of coupled harmonic oscillators. We also briefly discuss the method to evaluate entanglement entropy (EE) from a pure Gaussian ground state^39^. In the section titled *Results*, we provide details of the numerical and analytical tools in support of the observed QPT in the model. In the section titled *Discussions and Future Outlook*, we provide details of the calculation of our model Hamiltonian from an interacting fermionic theory and discuss possible applications to condensed matter systems.

## Model and Setup

In this section, we discuss the model Hamiltonian and provide essential steps to obtain entanglement entropy. We also show how our model Hamiltonian can be mapped to a system of coupled harmonic oscillators. Finally, we discuss the real-time method to evaluate the ground state reduced density matrix for the model Hamiltonian and the approach to evaluate the ground state entanglement entropy.

### Model Hamiltonian

We consider the following two dimensional Hamiltonian:

1 $$H=\frac{1}{2}\int {d}^{2}{\bf{r}}[|\hat{{\rm{\Pi }}}({\bf{r}}){|}^{2}+|{\rm{\nabla }}\hat{{\rm{\Phi }}}({\bf{r}}){|}^{2}+\frac{\varepsilon }{{\kappa }^{2}}|{{\rm{\nabla }}}^{2}\hat{{\rm{\Phi }}}({\bf{r}}){|}^{2}+\frac{\tau }{{\kappa }^{4}}|{{\rm{\nabla }}}^{3}\hat{{\rm{\Phi }}}({\bf{r}}){|}^{2}],$$

where $\hat{{\rm{\Pi }}}({\bf{r}})$ is the conjugate momenta of the massless Bosonic scalar field $\hat{{\rm{\Phi }}}({\bf{r}})$, *ε* and *τ* are dimensionless constants (and here it take values either 0 or 1), *κ* has the dimension of wave number which sets the scale for the deviation from Lorentz invariance and the speed of propagation is set to unity, ${\nabla }^{2}$ is the two dimensional Laplace operator, ${\nabla }^{4}$ and ${\nabla }^{6}$ are the higher order spatial operators of the circular coordinates ${\bf{r}}(=r,\theta )$ and $c=\hslash =1$. The equal time canonical commutation relation between fields is given by,

2 $$[\hat{{\rm{\Phi }}}({\bf{r}}),\hat{{\rm{\Pi }}}({\bf{r}}^{\prime} )]=i\,{\delta }^{2}({\bf{r}}-{\bf{r}}^{\prime} )=\frac{i}{r}\delta (r-r^{\prime} )\delta (\theta -\theta ^{\prime} )$$

Before going to the details, we briefly discuss some of the salient features of the model Hamiltonian in Eq. (1): (i) The above Hamiltonian corresponds to free field with a non-linear dispersion relation between frequency *ω* and wave number *k* via, ${\omega }^{2}={k}^{2}+\varepsilon \,{k}^{4}/{\kappa }^{2}+\tau \,{k}^{6}/{\kappa }^{4}$. (ii) It is well-known that ${\nabla }^{4}$ term with non-linear terms lead to classical Lifshitz transitions^40,41^. Here, we show that ${\nabla }^{6}$ terms or NNN coupling drives QPT^42^. (iii) It is important to note the differences between quantum Lifshitz transitions and our case. In the quantum Lifshitz transition, scalar field theory has dynamical critical exponent *z* = 2 and the dispersion relation is ${\omega }^{2}={k}^{2}+\varepsilon \,{k}^{4}/{\kappa }^{2}$ ^2,12,23^. However, in our case, the dynamical critical exponent is *z* = 3 and ${\omega }^{2}={k}^{2}+\varepsilon \,{k}^{4}/{\kappa }^{2}+\tau \,{k}^{6}/{\kappa }^{4}$ is the dispersion relation. (iv) It is known that quantum fluctuations play an important role in lower dimensions, and almost invariably, destroy long-range order. In the classical systems, Mermin-Wagner Theorem^43–45^ precludes true long-range order in the thermodynamic limit at non-zero temperature in one and two dimensions. We show that, in two dimensions, NNN coupling introduces instability and drive quantum fluctuations leading to QPT.

### Mapping of higher order spatial Hamiltonian to a system of coupled harmonic oscillators

Using the following ansatz:

3a $$\hat{{\rm{\Pi }}}({\bf{r}})=\sum _{m=-{\rm{\infty }}}^{{\rm{\infty }}}\frac{{\hat{{\rm{\Pi }}}}_{m}(r)}{\sqrt{\pi r}}\cos m\theta ,$$

3b $$\hat{{\rm{\Phi }}}({\bf{r}})=\sum _{m=-{\rm{\infty }}}^{{\rm{\infty }}}\frac{{\hat{\phi }}_{m}(r)}{\sqrt{\pi r}}\cos m\theta ,$$

we expand the two dimensional real scalar fields in Hamiltonian (1) (it is important to note that the Hamiltonian has same set of eigenvalues if we expand in sine function. For the case of complex scalar fields, one has to expand it in terms of exponential function). In the above expressions (3), *m* is the angular momentum quantum number, *r* and *θ* are the radial and angular coordinates, respectively.

The canonical commutation relation between the new rescaled fields is

4 $$[{\hat{\phi }}_{m}(r),{\hat{{\rm{\Pi }}}}_{m{\rm{^{\prime} }}}(r{\rm{^{\prime} }})]=i\,\delta (r-r{\rm{^{\prime} }})\,{\delta }_{mm{\rm{^{\prime} }}}$$

**Figure 1:**
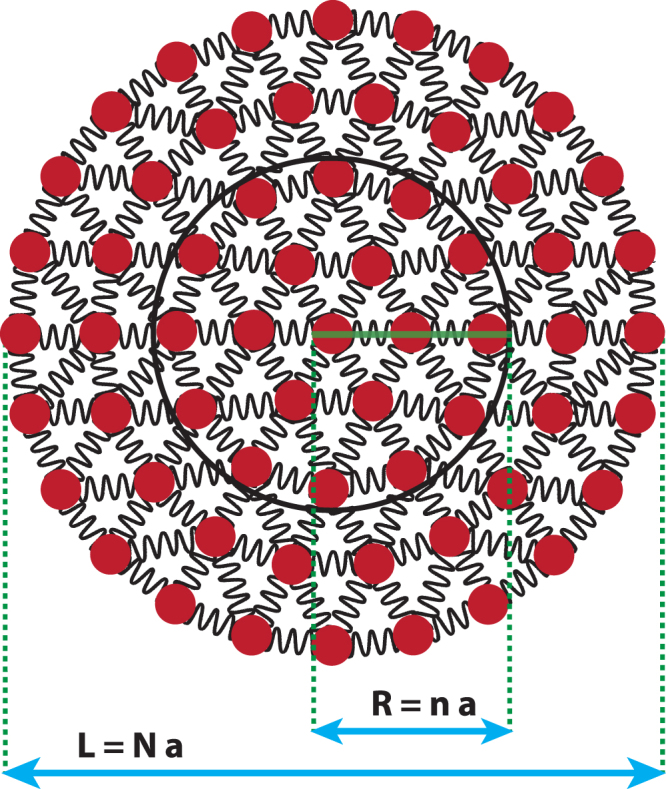
A pictorial representation of the radial lattice chain. The brown dots are *N* harmonic lattice sites arranged radially, *a* is the lattice parameter which acts as an ultraviolet cut-off and *L* = *Na* is the total length of the system which act as an infrared cut-off. In this arrangement, we trace over first (*N* − *n*) oscillators and evaluate reduced density matrix *ρ*_red_ and EE. For the nearest-neighbour and NN coupling, it can be seen that entropy scales as area of the boundary *n*, however, in the case of NNN coupling entropy scales non-trivially with *n*.

To map the model Hamiltonian (1) to a system of coupled harmonic oscillators; we perform integration over the angular coordinate *θ* by invoking orthogonal properties of cosine functions. We then discretize all radial derivative terms using central difference discretization scheme^46^. More explicitly, we use infrared cut-off as *L* = *Na*, where *N* is the number of Lattice points and *a* is the ultraviolet cut-off. In the discretized version, the dimensionless scalar fields are expressed as ${\hat{{\rm{\pi }}}}_{m,j}=a\,{\hat{{\rm{\Pi }}}}_{m}(ja)$, ${\hat{\phi }}_{m,j}={\hat{\phi }}_{m}(ja)$, and radial distance is *r* = *ja*. Hence, the form of Hamiltonian and commutation relation in Eqs (1) and (4), become (for more details see supplementary material),

5 $$H(P)=\frac{1}{2a}\sum _{m=-{\rm{\infty }}}^{{\rm{\infty }}}\sum _{i,j=1}^{N}[{\hat{\pi }}_{m,i}^{2}{\delta }_{ij}+{\hat{\phi }}_{m,i}\,{K}_{ij}(P,m)\,{\hat{\phi }}_{m,j}]$$

6 $$[{\hat{\phi }}_{m,i},{\hat{{\rm{\pi }}}}_{m{\rm{^{\prime} }}},j]=i{\delta }_{mm{\rm{^{\prime} }}}{\delta }_{ij}$$

7 $$\,{\rm{where}}\,P=\frac{1}{{(\kappa a)}^{2}}$$

is the coupling parameter that determines the extend of deviation from the linear dispersion relation, and *K*_*ij*_ is the real symmetric matrix which contains all the coupling terms— the nearest, the next-to-next nearest (NN), and NNN coupling, more specifically it includes the coefficients of *P* and *P* ^2^ (see supplementary material for more details about elements of the *K*_*ij*_ matrix). As can be seen, the Hamiltonian (5) corresponds to N-coupled harmonic oscillators. We use an open boundary condition, ${\hat{\phi }}_{m,N+1}=0$ to calculate EE and other relevant quantities. Fig. (1) provides a pictorial representation of the radial harmonic lattice.

The EE of the (*N* − *n*) traced oscillators is given by^47^,

8 $$S=-{\rm{Tr}}({\rho }_{{\rm{red}}}\,\mathrm{log}\,{\rho }_{{\rm{red}}})$$

where *ρ*_red_ is the reduced density matrix obtained by integrating over the (*N* − *n*) oscillators. The entanglement spectra of the entanglement Hamiltonian *h*_E_ is given by^29^,

9 $${h}_{{\rm{E}}}=-\mathrm{log}\,{\rho }_{{\rm{red}}}\mathrm{.}$$

### Evaluation of the entanglement entropy for the ground state

As mentioned earlier, reduced density matrix (*ρ*_*red*_) provides information about the strength of the quantum correlations across different regions. *ρ*_*red*_ is evaluated by tracing out the quantum degrees of freedom associated with the scalar order parameter inside a two dimensional region of radius *R*. *ρ*_red_ can be computed semi-analytically from Hamiltonian (5).

The procedure to obtain the entanglement entropy is similar to the one discussed in refs^39,42^. We assume that the quantum state corresponding to the Hamiltonian of the *N*-harmonic oscillator system is the ground state i. e. ${\psi }_{{\rm{G}}S}({x}_{1},{x}_{2},\cdots ,{x}_{n},{t}_{1},{t}_{2},\cdots ,{t}_{N-n})$. *ρ*_*red*_ is obtained for *n* oscillators by tracing over (*N* − *n*) of the *N* oscillators. von Neumann entropy quantifies the ground state entanglement of a bipartite system via $S=-Tr\,({\rho }_{{\rm{red}}}\,log{\rho }_{{\rm{red}}})$ ^39,48^.

The ground state wave function of the above Hamiltonian in Eq. (5) is given by,

10 $${\psi }_{{\rm{GS}}}(X)={(\frac{Det{\rm{\Lambda }}}{{\pi }^{N}})}^{\mathrm{1/4}}\exp (-{X}^{T}\mathrm{.}{\rm{\Lambda }}\mathrm{.}X/\mathrm{2)}$$

where

$${\rm{\Lambda }}=\sqrt{K}=(\begin{array}{cc}A & B\\ {B}^{T} & C\end{array})$$ and A, B, C are the sub matrices of Λ matrix with dimensions $n\times n,n\times (N-n),$ and $(N-n)\times (N-n)$ respectively such that $N > n$ and $X={({x}_{1},{x}_{2},\ldots {x}_{n},{t}_{1},{t}_{2},\ldots {t}_{N-n})}^{T}$. The ground state density matrix is

11 $${\rho }_{{\rm{GS}}}(X,X^{\prime} )={\psi }_{{\rm{GS}}}(X){\psi }_{{\rm{GS}}}^{\ast }(X^{\prime} )={\psi }_{{\rm{GS}}}({x}_{1},{x}_{2},\cdots ,{x}_{n},{t}_{1},{t}_{2},\cdots ,{t}_{N-n})\,{\psi }_{{\rm{GS}}}^{\ast }({x}_{1},\cdots ,{x}_{n},{t}_{1},{t}_{2},\cdots ,{t}_{N-n})$$

The reduced density matrix (${\rho }_{{\rm{red}}}$) for the ground state, is obtained by tracing over the first (*N* − *n*) of *N* oscillators of the pure density matrix *ρ*_GS_

12 $${\rho }_{{\rm{r}}ed}(x,x^{\prime} )=\int dt\,{\rho }_{GS}(X,X^{\prime} )$$

13 $$=\int (\prod _{i\mathrm{=1}}^{N-n}d{t}_{i}){\psi }_{{\rm{GS}}}({x}_{1},{x}_{2},\ldots {x}_{n},{t}_{1},{t}_{2},\ldots {t}_{N-n}){\psi }_{{\rm{GS}}}^{\ast }({x}_{1^{\prime} },{x}_{2^{\prime} },\ldots {x}_{n^{\prime} },{t}_{1},{t}_{2},\ldots {t}_{N-n})$$

where $x={({x}_{1},{x}_{2},\ldots ,{x}_{n})}^{T}$ and $t={({t}_{1},{t}_{2},\ldots ,{t}_{n})}^{T}$. The integral in Eq. (12) can be evaluated explicitly and can be written as,

14 $${\rho }_{{\rm{red}}}(x,x^{\prime} )\sim \exp [-({x}^{T}\mathrm{.}{\rm{\Gamma }}\mathrm{.}x+{x^{\prime} }^{T}\mathrm{.}{\rm{\Gamma }}\mathrm{.}x^{\prime} )/2+{x}^{T}\mathrm{.}{\rm{\Omega }}\mathrm{.}x^{\prime} ]$$

where Ω and Γ are defined as

15a $${\rm{\Omega }}=\frac{1}{2}{B}^{T}{A}^{-1}B,$$

15b $${\rm{\Gamma }}=C-{\rm{\Omega }}$$

Let ${{\rm{\Gamma }}}_{D}$ and V be the diagonal and orthogonal matrices of Γ. Performing the transformation $x={V}^{T}{{\rm{\Gamma }}}_{D}^{-\mathrm{1/2}}y$ then ${\rm{\Omega }}\to {\rm{\Omega }}^{\prime} ={{\rm{\Gamma }}}_{D}^{-\mathrm{1/2}}V{\rm{\Omega }}{V}^{T}{{\rm{\Gamma }}}_{D}^{-\mathrm{1/2}}$, the reduced density matrix becomes,

16 $${\rho }_{{\rm{red}}}(y,y^{\prime} )\sim \exp [-(y\mathrm{.}y+y^{\prime} \mathrm{.}y^{\prime} \mathrm{)/2}+{y}^{T}\mathrm{.}{\rm{\Omega }}^{\prime} \mathrm{.}y^{\prime} ]$$

Rewriting *y* = *Wz*, where W is an orthogonal matrix such that Ω′ is diagonal in W basis, Eq. (16) becomes,

17 $${\rho }_{{\rm{red}}}(z,z^{\prime} )\sim \prod _{i\mathrm{=1}}^{n}\exp [-({z}_{i}^{2}+{z^{\prime} }_{i}^{2})/2+{{\rm{\Omega }}^{\prime} }_{i}{z}_{i}{z^{\prime} }_{i}]$$

where ${{\rm{\Omega }}^{\prime} }_{i}$ is an eigenvalue of Ω′. von Neumann entropy of the reduced density matrix (*ρ*_*red*_) is given by,

18 $${S}_{m}=\sum _{i\mathrm{=1}}^{n}{S}_{m,i}({\xi }_{i})$$

where

19a $${S}_{m,i}(\xi )=\,\mathrm{log}\,\mathrm{(1}-{\xi }_{i})-\frac{{\xi }_{i}}{1-{\xi }_{i}}\,\mathrm{log}\,{\xi }_{i},$$

19b $${\rm{and}}\,{\xi }_{i}=\frac{{{\rm{\Omega }}}_{i}\text{'}}{1+\sqrt{1-{{\rm{\Omega }}}_{i}{\text{'}}^{2}}}$$

The entanglement entropy, *S*, is computed by summing over all *m* modes as

20 $$S={S}_{m\mathrm{=0}}+2\sum _{m\mathrm{=1}}^{\infty }{S}_{m}$$

where ${S}_{m\mathrm{=0}}$ is the value of EE for *m* = 0 and all other *S*_*m*_ values are multiplied by a degeneracy factor 2.

## Results

In this section, we evaluate the physical quantities for the model Hamiltonian as discussed in the section titled *Model Hamiltonian*. In the first part, we obtain the numerical tools — entanglement entropy and entanglement spectrum — to confirm the evidence of QPT. In the second part, we use analytical tools like quantum ground state fidelity and gap in the energy spectra to identify the cause of QPT.

### Numerically evaluated tools

We compute the entanglement entropy numerically for the discretized Hamiltonian presented in Eq. (5). The computations are done using Matlab for the lattice size *N* = 600, $10\le n\le 590$ and the error in computation is 10^−8^. More specifically, we choose the following cases for the numerical evaluation of two dimensional real-space entanglement entropy;

(I) *τ* = 0, *ε* = 1. — The *K*_*ij*_ matrix contains both nearest neighbour and NN coupling corresponding dispersion relation is *ω* ^2^ = *k* ^2^ + *k* ^4^/*κ* ^2^.
(II) *τ* = 1, *ε* = 0. — The *K*_*ij*_ matrix contains nearest neighbour, NN, and NNN coupling and the corresponding dispersion relation is *ω* ^2^ = *k* ^2^ + *k* ^6^/*κ* ^4^.
(III) *τ* = 0, *ε* = ±1. — The *K*_*ij*_ matrix contains nearest neighbour, NN, and NNN coupling and the corresponding dispersion relation is *ω* ^2^ = *k* ^2^ ± *k* ^4^/*κ* ^2^ + *k* ^6^/*κ* ^4^.

For case (I), like in canonical scalar field, the entanglement entropy scales linearly as *n* for all values of *P*. Hence, we will not present the results in the main text (see supplementary material for more details). The results for case (III) are similar to case (II), hence, we only present the results for case (II).

**Figure 2:**
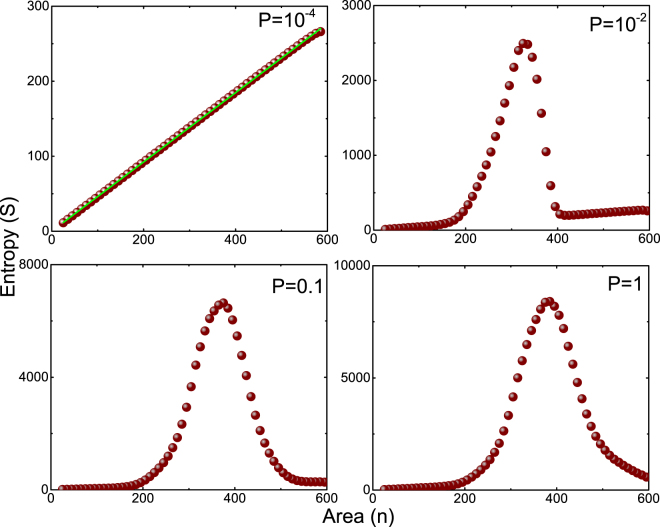
Plots of two dimensional vacuum entanglement entropy (*S*) versus area (*n*) for case II ($\tau =1$, $\varepsilon =0$) is shown for different values of *P*. The brown dots are the numerical output data points and the green line is the best linear fit.

In Fig. (2), we have plotted *S* versus *n* for different values of the coupling constant *P*. The following points are interesting to note: First, the entropy profile changes as the coupling strength *P* is increased. In the case of $P={10}^{-4}$, the entropy is linearly related to *n*, however, as *P* increases the entropy-area linear relation is broken and the entropy changes by an order. It is interesting to see in the case of higher values of *P*, for large values of *n* the entropy scales linearly with area up to certain value of *n* and it is highly non-linear and for small values of *n* the entropy scales linearly with area. Second, it is known that the area-law is valid for gapped systems^21,49^. It is important to note that the addition of NNN coupling make the system gap-less and the area-law is violated. Third, the area-law violation presented here is a generic feature for the Hamiltonian (5) containing NNN coupling and it is intrinsic to gap-less quantum Hamiltonian. In other words, the area-law violation exists for all values of lattice points *N*’s (see supplementary material for more details). Fourth, the area-law is satisfied for case (I), dispersion relation is ${\omega }^{2}={k}^{2}+{k}^{4}/{\kappa }^{2}$ and dynamical critical exponent is $z=2$, which is consistent with the analysis reported in ref.^24^. The expression for entanglement entropy is $S=a\,n+b\,\mathrm{log}\,n$, where *a* and $b$ are some arbitrary constants depends on the nature of partition used in the bipartite set-up (for more details see Figs (1) and (2) in supplementary material and in our analysis, $a=1.39$ and $b=0.038$).

The model Hamiltonian in Eq. (1) was first studied in three dimensional space and concluded with the following salient remarks^42^: (i) EE violates area-law in three dimensional space. (ii) NNN coupling term is responsible for the change in the behaviour of EE and thus changes the ground system properties of the system at QCP. (iii) Like in the two dimensional case, entanglement entropy in three dimensions scales linearly with *n* for small values of *P*. However, for large values of *P*, it scales inversely. In both cases, the study of EE reveals that the violation of area-law is an inherent property of the Hamiltonian in Eq. (5) with NNN coupling. It is possible to identify the following scaling transformations $r\to {r}{{e}}^{-\iota },\hat{{\rm{\Phi }}}\to {\rm{\Phi }}{e}^{\iota },\kappa \to \kappa {e}^{\iota }$, where $\iota$ is the scaling parameter such that Hamiltonian remains invariant. It is interesting to note that we have evaluated EE in one dimension where the entropy remains a constant, that is, area-law is followed. Hence, two dimension is the critical dimension for observing the change in the behaviour of EE.

The above comparison and results conclusively show that NNN coupling lead to different quantum phases. To firmly identify what causes the change in the entropy by an order, in the rest of this work, we use three quantifying measures — entanglement spectrum, ground state fidelity, and many-body ground state energy — and show that this is indeed QPT. It is important to note that the first measure is numerical while the other two measures are analytical.

#### Spectra of entanglement Hamiltonian

Reduced density matrix contains complete information about quantum entanglement, however, entanglement entropy being scalar may not provide complete information^30^. Entanglement Hamiltonian is an imaginary system that describes the correlations of the ground state. While it cannot be measured directly, it is related to the statistics of the fluctuations in the lattice. Entanglement Hamiltonian $({h}_{{\rm{E}}})$ is defined as ${h}_{E}=-\mathrm{log}\,{\rho }_{{\rm{red}}}$ and plays the role of $\beta H$ in thermodynamic systems^30^. It has been shown that the largest and the second largest eigenvalues of *h*_E_ forms a gap at the QCP^30^ and widely considered as a tool for quantifying QCP^32–34^. In the same spirit, we evaluate the first two largest eigenvalues of *h*_E_ and verify the formation of gap at QCP.

**Figure 3:**
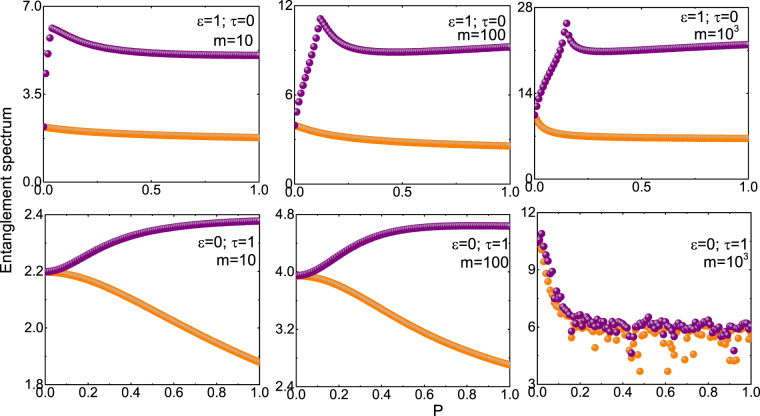
Plots show the variation of the largest (orange) and second largest (purple) eigenvalues of the entanglement spectrum as a function *P* for different *m* values. The top panel is for case I ($\varepsilon =\mathrm{1,}\,\tau =\mathrm{0,}\,n=300$) while the bottom panel is for case II ($\varepsilon =\mathrm{0,}\,\tau =\mathrm{1,}\,n=300$).

In Fig. (3), largest and the second largest eigenvalue of *h*_E_ are plotted for different coupling parameter *P* for NN and NNN coupling, from which we infer the following: (i) In the case of NN coupling, the largest and the second largest eigenvalues of *h*_E_ have a gap for all values of the coupling constant except at $P=0$. (ii) In the case of NNN coupling, the largest and the second largest eigenvalues of *h*_E_ is degenerate for $P < 0.17$, however, above the critical point $P=0.17$ the two eigenvalues are non-degenerate. (iii) The presence of the entanglement gap at a finite *P* provides an evidence of quantum critical point at $P=0.17$. It is also interesting to note that it is at the same point that the overlap function also shows a sharp change^34^.

**Figure 4:**
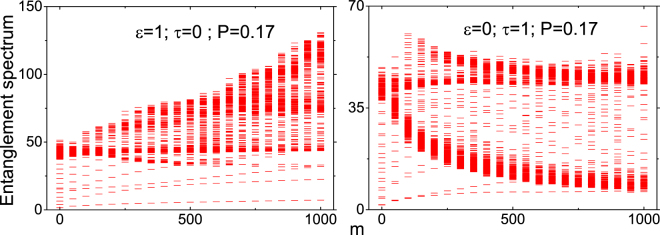
Plot shows full entanglement spectrum of *h*_E_ for case I (left plot) and case II (right plot) respectively for $n=300$ and $N=600$. The red lines correspond to different entanglement energy levels.

Figure (4) contains all the eigenvalues of the entanglement Hamiltonian *h*_E_ for the NN and NNN coupling. It is interesting to note that in Case I, bulk of the spectrum is continuous. This has to be contrasted with case II where only the edges are continuous. Similar behaviour spectrum is also seen in the fractional quantum hall states^30^ and spin chains in momentum space^35^. The fingerprint of QPTs can be confirmed by the non-collapsing of entanglement gap — the entanglement energy gap between the lowest and the highest entanglement part in the subsystem^30,35,37,50^. In other words, NNN coupling term brings a gap — that is absent in NN coupling case — suggesting that QPT is triggered by the linear scalar field. This gap is generally termed as the entanglement gap or Schmidt gap which is considered here as an order parameter to diagnose QPT^32,33^.

### Analytically evaluated tools

In this subsection we discuss the analytical results to confirm the evidence for violation of area-law observed in Hamiltonian (1). We use two main tools such as ground state overlap function or quantum fidelity and gap in the spectrum to verify the phase transitions in Bosonic Hamiltonian with NNN couplings.

#### Ground state Overlap function and Energy gap

The ground state overlap function (Quantum fidelity) is defined for the ground state as $F=\langle {\psi }_{{\rm{GS}}}(P+\delta P)|{\psi }_{{\rm{GS}}}(P)\rangle$, where $\delta P$ is the infinitesimal change in the value of $P$ ^51,52^. It has been shown that sudden change in the overlap function indicates quantum criticality and hence is a good quantitative measure to identify QCP^53–55^.

**Figure 5:**
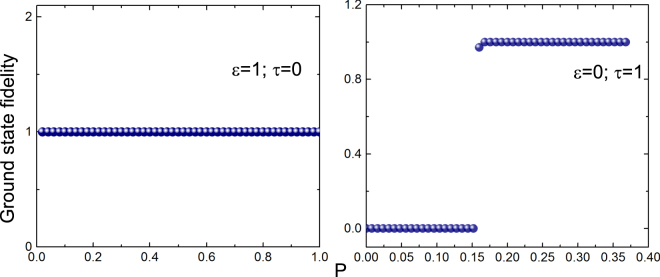
Left plot is the ground state fidelity for case I ($\varepsilon =\mathrm{1,}\,\tau =0$) while the right plot is the ground state fidelity for case II ($\varepsilon =\mathrm{0,}\,\tau =1$). All plots are for $m=0$ and $\delta P={10}^{-8}$.

In Fig. (5), overlap function is plotted for different coupling parameter *P* for the NN and NNN coupling terms, from which we infer the following: (i) With the NN coupling terms the overlap function is always close to unity implying that the ground state many body wave functions for *P* and $P+\delta P$ are identical. This is consistent with the results of entanglement entropy that the entropy linearly scales with *n* for all values of *P*. (ii) With the NNN coupling term, the overlap function shows a sudden discontinuity close to $P=0.17$. For $P > 0.17$, the ground state wave functions (as in the case of NN coupling) for *P* and $P+\delta P$ are identical, however, for $P < 0.17$ ground state wave functions for *P* and $P+\delta P$ are orthogonal. This is also consistent with the results of entanglement entropy that entropy is non-linearly related to *n*. (iii) While the overlap function signals that the ground state wave function is not identical for *P* close of $0.17$, entanglement entropy shows non-linear behaviour even at $P=0.01$. This also signals that the NNN coupling term leads to instability and leads to different phases.

It has been suggested that the signature of QPT is also encoded in the many-body ground state of the system^19–21^. At quantum criticality, ground state energy of the Hamiltonian $H(P)$ has a gap which is related to the correlation length of the system. In other words, gap becomes singular — developing infinite correlation length across the critical point^19–21^.

**Figure 6:**
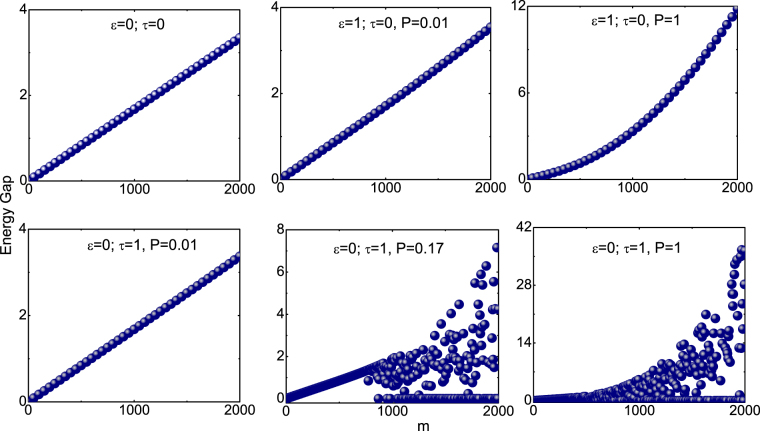
Plots of energy gap— separation between the ground state and first excited state— of $H(P)$ as a function of *m*. The first figure in the top panel for the nearest neighbouring term and the remaining two figures for case I $(\varepsilon =\mathrm{1,}\,\tau =0)$ while the bottom panel is for case II $(\varepsilon =\mathrm{0,}\,\tau =1)$.

In Fig. (6), the energy gap of Hamiltonian $H(P)$— energy separation between the ground and first excited states— is plotted for different values of *m* for the NN and NNN coupling terms (at constant *P*), from which we infer the following: (i) For the NN coupling term, the energy gap vanishes only for $m=\mathrm{0,}\,P=0$. One can infer that only one zero mode is present, that is there is a finite energy separation between the ground and first excited states for any finite values of *P*. (ii) In the case of NNN coupling term, above the critical values of $P=0.17$, several values of *m* have zero energy gap or zero mode. One infers that multiple zero modes exist for all coupling constants above the critical $P=({P}_{c}=\mathrm{0.17)}$. (iii) From the linear behaviour of the energy versus *m* confirms the validity of area-law in the case of NN coupling is because of the absence of zero mode at any non-zero *P* values. The accumulation of large number of zero modes at and above the critical point $P=0.17$ characterise the violation of area-law in the case of NNN coupling. (iv) Interestingly, the violation of area-law can be attributed to high angular modes as one can see it in Fig. 6 while after some critical angular modes its contribution decreases, hence we can use a cut-off while computing EE numerically. It is important to note that this critical point is different from the point in which the entropy-area law is violated $(P\simeq {10}^{-2})$. In other words, entanglement entropy-area violation signals a drastic change in the ground state behaviour. Our model is yet another example of violation of area law in higher dimensions purely by the NNN coupling term in the Hamiltonian or the accumulation of large number of zero modes at any non-zero *P* values. More explicitly, the presence of multiple zero modes lead to the violation of entropy-area relation and drives QPT^19–21^.

## Discussions and Future Outlook

### What causes the quantum phase transition?

**Figure 7:**
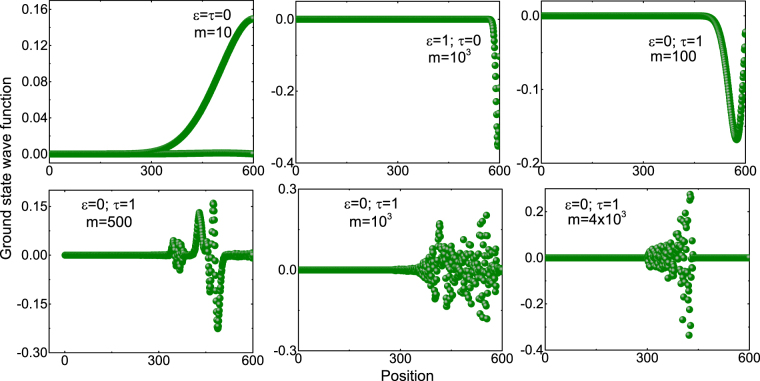
Plots of ground state wave function versus radial distance of lattice for nearest neighbour (top-left), NN coupling term (top cent-er), and NNN coupling terms for $n=300$ and $P=1$.

As we have shown explicitly, NNN coupling term leads to QPT. It is expected that QPT should be accompanied by a fundamental change in the ground state properties of the system. To go about understanding this, in Fig. 7, we have plotted the ground state wave function of the system as a function of position (lattice point). For small *m*, the wave-function peaks near the boundary, however, for higher *m* the wave-function is more dispersed and peaks at the centre.

Recalling the results plotted in Fig. 6, above the critical point $P=0.17$, several *m* have zero modes and the wave-function peaks at the centre. The following physical picture emerges: For small *P*, there exists only one ($m=0$) zero mode, however, as *P* increases the number of zero modes increase. This constitutes a fundamental change in the behaviour of the system and hence leading to a phase transition. It is quite clear that for the case of NNN coupling, the ground state wave function is changing for each *m* values and contribution is coming mainly from the bulk of the system. This change in the wave function is responsible for the accumulation of large number of zero modes in the system see Fig. 6 for each values of *m*. Hence, the phase transition reported in this work is purely driven by NNN coupling and large number of zero modes at any non-zero *P* values implies the presence of degenerate ground states and triggers QPT— system becomes gap-less^19–21^.

A qualitative understanding of excitation of higher modes (for $P > 0.17$) can be understood using the quantum mechanical model of a particle in a 2-dimensional box of length *L* ^42^. It was explicitly shown that

21 $$\frac{{\rm{G}}{\rm{r}}{\rm{o}}{\rm{u}}{\rm{n}}{\rm{d}}\,{\rm{s}}{\rm{t}}{\rm{a}}{\rm{t}}{\rm{e}}\,{\rm{e}}{\rm{n}}{\rm{e}}{\rm{r}}{\rm{g}}{\rm{y}}\,{\rm{e}}{\rm{i}}{\rm{g}}{\rm{e}}{\rm{n}}{\rm{v}}{\rm{a}}{\rm{l}}{\rm{u}}{\rm{e}}\,{\rm{f}}{\rm{o}}{\rm{r}}\,{\rm{c}}{\rm{a}}{\rm{n}}{\rm{o}}{\rm{n}}{\rm{i}}{\rm{c}}{\rm{a}}{\rm{l}}\,{\rm{s}}{\rm{c}}{\rm{a}}{\rm{l}}{\rm{a}}{\rm{r}}\,{\rm{f}}{\rm{i}}{\rm{e}}{\rm{l}}{\rm{d}}}{{\rm{G}}{\rm{r}}{\rm{o}}{\rm{u}}{\rm{n}}{\rm{d}}\,{\rm{s}}{\rm{t}}{\rm{a}}{\rm{t}}{\rm{e}}\,{\rm{e}}{\rm{n}}{\rm{e}}{\rm{r}}{\rm{g}}{\rm{y}}\,{\rm{e}}{\rm{i}}{\rm{g}}{\rm{e}}{\rm{n}}{\rm{v}}{\rm{a}}{\rm{l}}{\rm{u}}{\rm{e}}\,{\rm{f}}{\rm{o}}{\rm{r}}\,{\rm{c}}{\rm{a}}{\rm{s}}{\rm{e}}\,{\rm{I}}{\rm{I}}\,}={[\frac{L\kappa }{\pi }]}^{4}$$

Equation (21) implies that, for $\kappa  < \pi /L$, the ground state energy eigenvalue for case II is higher compared to that of canonical scalar field. With increasing *P* (or decreasing $\kappa$), system readjusts in such a way that the ground state energy of the system increases. In other words, the cross-over of the dispersion relation catalyses *larger population* of higher energy quantum modes.

### Plausible implications in Condensed matter physics: Deriving spatial higher derivative Hamiltonian from Hubbard- Stratonovich transformation

An interesting fact about the Hamiltonian (5) is that it can be derived from Hubbard- Stratonovich transformation (HST)— Bosonization of interacting fermions or transforming the partition function of a system of interacting fermions into the partition function of noninteracting Bosons. We briefly sketch details of the modified nonlinear dispersion relations from HST for the case of $\mathrm{(2}+\mathrm{1)}$ dimensions^56^.

The Euclidean action, ${{\mathscr{S}}}^{F}$, for interacting electrons is,

22 $${{\mathscr{S}}}^{F}=\int d{\tau }_{E}\,{d}^{2}x\,[\sum _{i=\uparrow ,\downarrow }{\psi }_{i}^{\dagger }({{\rm{\partial }}}_{{\tau }_{E}}+\frac{{{\rm{\nabla }}}^{2}}{2{\mathscr{M}}})\,{\psi }_{i}-g{\psi }_{\uparrow }^{\dagger }{\psi }_{\downarrow }^{\dagger }{\psi }_{\downarrow }{\psi }_{\uparrow }]$$

and the partition function is

23 $$Z=\int D[{\psi }^{\dagger }\psi ]{e}^{-{{\mathscr{S}}}^{F}}$$

where $\psi$ is the complex fermionic field, ${\tau }_{E}$ is the Euclidean time, ${\bf{x}}$ is the two dimensional space vector, ${\mathcal M}$ is the mass of electron and $g$ is the coupling constant. The interaction term in the above action can be decoupled by using HST which is given by,

24 $$\begin{array}{c}\exp \,[\displaystyle -\int d{\tau }_{E}\,{d}^{2}{\bf{x}}(g{\psi }_{\uparrow }^{\dagger }{\psi }_{\downarrow }^{\dagger }{\psi }_{\downarrow }{\psi }_{\uparrow })]=\displaystyle \frac{1}{{\mathscr{N}}}\int D[{{\rm{\Delta }}}^{\ast }{\rm{\Delta }}]\exp [\int d{\tau }_{E}\,{d}^{2}x(\frac{1}{g}{|{\rm{\Delta }}|}^{2}-{\rm{\Delta }}{\psi }_{\uparrow }^{\dagger }{\psi }_{\downarrow }^{\dagger }-{{\rm{\Delta }}}^{\ast }{\psi }_{\uparrow }{\psi }_{\downarrow })]\\ \,\,\,\,=\,\displaystyle \frac{1}{{\mathscr{N}}}\int D[{{\rm{\Delta }}}^{\ast }{\rm{\Delta }}]\exp [\int d{\tau }_{E}\,{d}^{2}x(\frac{1}{g}{|{\rm{\Delta }}-g{\psi }_{\uparrow }{\psi }_{\downarrow }|}^{2}-g\,{\psi }_{\uparrow }^{\dagger }{\psi }_{\downarrow }^{\dagger }{\psi }_{\downarrow }{\psi }_{\uparrow })]\end{array}$$

where ${\mathscr{N}}$ is the normalisation factor that arises after the integration over auxiliary complex field ${\rm{\Delta }}$. The action (22) can be written in the decoupled form after HST is,

25 $${{\mathscr{S}}}_{HS}^{F}=\int d{\tau }_{E}\,{d}^{2}x[\sum _{i=\uparrow ,\downarrow }{\psi }_{i}^{\dagger }({{\rm{\partial }}}_{{\tau }_{E}}+\frac{{{\rm{\nabla }}}^{2}}{2{\mathscr{M}}}){\psi }_{i}-\frac{1}{g}{|{\rm{\Delta }}|}^{2}+{\rm{\Delta }}{\psi }_{\uparrow }^{\dagger }{\psi }_{\downarrow }^{\dagger }+{{\rm{\Delta }}}^{\ast }{\psi }_{\uparrow }{\psi }_{\downarrow }]$$

and the partition function is

26 $$Z=\int D[{\psi }^{\dagger }\psi ;{{\rm{\Delta }}}^{\dagger }{\rm{\Delta }}]{e}^{-{{\mathscr{S}}}_{HS}^{F}}$$

It should be noted that the interacting fermionic theory becomes quadratic in both ${\rm{\Delta }}$ and $\psi$;

27 $${{\mathscr{S}}}_{HS}^{F}=\int d{\tau }_{E}\,{d}^{2}x[\sum _{i=\uparrow ,\downarrow }{\psi }_{i}^{\dagger }({G}^{-1}+{\rm{\Xi }}){\psi }_{i}-\frac{1}{g}{|{\rm{\Delta }}|}^{2}+{\rm{\Delta }}{\psi }_{\uparrow }^{\dagger }{\psi }_{\downarrow }^{\dagger }+{{\rm{\Delta }}}^{\ast }{\psi }_{\uparrow }{\psi }_{\downarrow }]$$

where we used

28 $${G}^{-1}=(\begin{array}{cc}{{\rm{\partial }}}_{{\tau }_{E}}-\frac{{(-i{\rm{\nabla }})}^{2}}{2{\mathscr{M}}} & 0\\ 0 & {{\rm{\partial }}}_{{\tau }_{E}}-\frac{{(i{\rm{\nabla }})}^{2}}{2{\mathscr{M}}}\end{array})\,{\rm{a}}{\rm{n}}{\rm{d}}\,\,\,{\rm{\Xi }}=(\begin{array}{cc}0 & {\rm{\Delta }}\\ {{\rm{\Delta }}}^{\ast } & 0\end{array}),$$

the space of these matrices are in the Nambu space. Performing integration over the fermionic fields using HST action^57^,

29 $$\begin{array}{ccc}Z & = & \displaystyle \int D[{\psi }^{\dagger }\psi ;\,{{\rm{\Delta }}}^{\dagger }{\rm{\Delta }}]\,{e}^{-{{\mathscr{S}}}_{HS}^{F}}\\  & = & \displaystyle \frac{1}{{\mathscr{N}}}\int D[{{\rm{\Delta }}}^{\dagger }{\rm{\Delta }}]\,Det[{G}^{-1}+{\rm{\Xi }}]\,\exp [-\int d{\tau }_{E}\,{d}^{2}{\bf{r}}\frac{1}{g}{|{\rm{\Delta }}|}^{2}]\\  & = & \displaystyle \frac{1}{{\mathscr{N}}}\int D[{{\rm{\Delta }}}^{\dagger }{\rm{\Delta }}]\,\exp [(\int d{\tau }_{E}\,{d}^{2}{\bf{r}}\,\frac{1}{g}|{\rm{\Delta }}{|}^{2})+Tr\,{\rm{l}}{\rm{o}}{\rm{g}}[{G}^{-1}+{\rm{\Xi }}]],\end{array}$$

where in the last step we have used the matrix trace property $Det\,({\rm{matrix}})=\exp (Tr\,\mathrm{log}\,[\,{\rm{matrix}}])$, $Det$, and $Tr$ refer to determinant and trace operation of a matrix, respectively.

The effective action for Bosonic theory is given by;

30 $$\begin{array}{ccc}{{\mathscr{S}}}_{eff} & = & (-\int d{\tau }_{E}\,{d}^{2}{\bf{r}}\,\frac{1}{g}{|{\rm{\Delta }}|}^{2})+Tr\,{\rm{l}}{\rm{o}}{\rm{g}}\,[{G}^{-1}+{\rm{\Xi }}]\\  & = & (-\int d{\tau }_{E}\,{d}^{2}{\bf{r}}\,\frac{1}{g}{|{\rm{\Delta }}|}^{2})-Tr\,{\rm{l}}{\rm{o}}{\rm{g}}\,{G}^{-1}-Tr(G{\rm{\Xi }}-\frac{1}{2}G\,{\rm{\Xi }}G{\rm{\Xi }}+\ldots )\end{array}$$

In the above trace expansion, all odd powers of the combination $G\,{\rm{\Xi }}$ are zero and the expansion of ${G}^{-1}$ around the high energy limit will bring the nonlinear dispersion relation like ${k}^{2}+\alpha {k}^{4}+{\alpha }^{2}{k}^{6}$, where *α* is some dimension-full constant. Hence, the dispersion relation of Eq. (1) is similar to the dispersion relation obtained from the effective Bosonic Hamiltonian of some interacting fermionic theory in the high energy limit. Normally, Bosonization obtained by using HST is in the low energy regime, but in this case it is done at the high energy limit and we study the effects of NNN interactions in the system. This modified dispersion relation is usually referred as “trans-Bogoliubov” dispersion relation^58^. Also, the Lorentz symmetry breaking term ${k}^{6}$ plays a fundamental role in the renormalization of the Feynman propagators in the high energy limit (for more details see the ref.^58^). Hence, the Hamiltonian (1) plays a crucial role in the case of strongly interacting systems and its effective action in the high energy limit.

### Future outlook of the spatially coupled radial Hamiltonian

Our analysis explicitly shows that NNN coupling term drives QPT by modifying dispersion relation and can play an important role in the high energy limit of the interacting Fermions. One possible strongly correlated condensed matter system that may be interest is high temperature superconductors (HTS). It is long-known that, in HTS, the coloumbic interactions between the electrons tend to make an anti-ferromagnetic arrangement of spins in the Copper Oxide planes and the magnetic transition is controlled by the weak coupling between the planes along the z-axis^59^.

To overcome the complexity of the interactions, let us consider Bosonic scalar field $\hat{{\rm{\Phi }}}$ in 3-dimensional cylindrical geometry such that the higher derivative terms contribute only in the 2-dimensional plane while the first derivative term contribute in all the three spatial dimensions. Repeating the analysis in 3-dimensions, it can be shown that the model in 3-dimensions has the same entropy profile as that in the 2-dimensional case (for detailed calculations, see section (IV-A) of the supplementary material).

**Figure 8:**
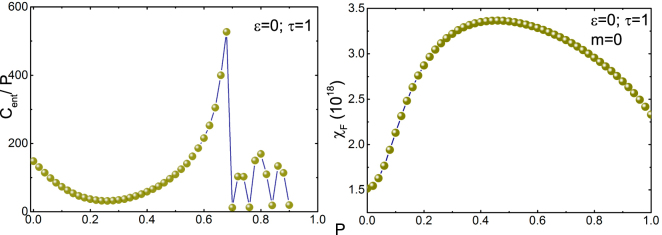
Left figure is the plot of entanglement specific heat per $P$ $({C}_{ent}/P=d{S}_{\varsigma \to \infty }/dP)$ versus $P$ obtained from the single copy entanglement entropy^63^ (${S}_{\infty }$ is the Rényi entropy having infinity as the Rényi index). Right figure is the plot of fidelity susceptibility $({\chi }_{F}=\displaystyle \mathop{lim}\limits_{\delta P\to 0}-2({\delta }^{2}/\delta {P}^{2}){\rm{l}}{\rm{o}}{\rm{g}}\,{P}^{2})$ as a function of $P$. We have taken lattice size of the $z$-axis to be 10 times more than the lattice size of the 2-dimensional surface, $N=600,\,n=300$ and $\delta P={10}^{-7}$.

One interesting feature is that the entanglement specific heat in Fig. 8 in our simplified model shows discontinuity at a particular value of $P$. This is indeed similar to the discontinuity of the specific heat measurement of the single crystals of YBa_2_Cu_3_O_7−δ_^60^. (See Sec. (IVB) of the supplementary material.)

Recently, treating entanglement as an analogue of energy, Brandão and Plenio showed that entanglement satisfy analogous first law of thermodynamics^61^. Once a complete analogy between entanglement and thermodynamics is build, it may be possible to understand the relation between the entanglement specific heat and the specific heat in thermodynamics. While the model proposed is a simple scalar order parameter, it can explain some of the crucial experimental measurements including the discontinuity^62^. Our goal is to include charge carriers in the model including NNN coupling term and explain other phenomena in the HTS. We hope to report this in the future.

## Electronic supplementary material

Supplementary Material
