# Supplementary material for: Evidence of quantum phase transition in real-space vacuum entanglement of higher derivative scalar quantum field theories
Source: Sci Rep. 2017 Nov 17;7:15774. doi: 10.1038/s41598-017-15858-9 (PMC5693898; doi:10.1038/s41598-017-15858-9)
Supplement: Supplementary file 1 — Supplementary Material [file 41598_2017_15858_MOESM1_ESM.pdf]

—Supplementary Material—

Evidence of quantum phase transition in real-space vacuum entanglement of higher derivative scalar quantum field theories

S. Santhosh Kumar[1*] and S. Shankaranarayanan[1,2†]
[1]*School of Physics, Indian Institute of Science Education and Research Thiruvananthapuram, CET Campus, Thiruvananthapuram 695 016, Kerala, India and*
[2]*Department of Physics, Indian Institute of Technology Bombay, Mumbai 400 076, Maharashtra, India*

The Supplementary material contains details about the interaction matrix elements that we have used to evaluate vacuum state entanglement entropy in two dimensional space. We plot the entanglement entropy for different lattice sites and show that the behaviour of entropy is independent of sites. We also include details of the entanglement entropy in cylindrical case and its implications for high temperature superconductivity. All notations used here are consistent with the main text of the manuscript.

I. THE INTERACTION MATRIX ELEMENTS IN TWO DIMENSIONAL SPACE

The 2− dimensional higher spatial derivative Hamiltonian is [1]

$$\hat{H} = \frac{1}{2}\int d^2\mathbf{r}\left[|\hat{\Pi}(\mathbf{r})|^2 + \left|\nabla\hat{\Phi}(\mathbf{r})\right|^2 + \frac{\epsilon}{\kappa^2}\left|\nabla^2\hat{\Phi}(\mathbf{r})\right|^2 + \frac{\tau}{\kappa^4}\left|\nabla^3\hat{\Phi}(\mathbf{r})\right|^2\right] \tag{1}$$

where $\hat{\Phi}$ is the massless scalar field and $\hat{\Pi}$ is its conjugate momenta, ϵ and τ are dimensionless constants, κ has the dimension of wave number and $\mathbf{r}(= r, \theta)$ is the circular polar coordinates. All computations are done by setting $\hbar = c = 1$.

The equal time canonical commutation relation is given by,

$$\left[\hat{\Phi}(\mathbf{r}), \hat{\Pi}(\mathbf{r}')\right] = i\,\delta^2(\mathbf{r}-\mathbf{r}') = \frac{i}{r}\delta(r-r')\delta(\theta-\theta') \tag{2}$$

We use the following ansatz to expand the real scalar fields in circular polar coordinates

$$\hat{\Pi}(\mathbf{r}) = \sum_{m=-\infty}^{\infty}\frac{\hat{\Pi}_m(r)}{\sqrt{\pi r}}\cos m\theta \tag{3a}$$

$$\hat{\Phi}(\mathbf{r}) = \sum_{m=-\infty}^{\infty}\frac{\hat{\varphi}_m(r)}{\sqrt{\pi r}}\cos m\theta, \tag{3b}$$

where m is the angular momentum quantum number. The canonical commutation relation between the new rescaled fields is

$$\left[\hat{\varphi}_m(r), \hat{\Pi}_{m'}(r')\right] = i\,\delta(r-r')\,\delta_{mm'} \tag{4}$$

Integration over the polar angle θ is carried out by invoking the orthogonal properties of the cosine function. We then apply the central difference scheme on a radial lattice having N lattice points with a is the lattice parameter. The Hamiltonian in eq. (1) becomes,

$$\hat{H} \;=\; \frac{1}{2a}\sum_{m=-\infty}^{\infty}\sum_{j=1}^{N}\left[\hat{\pi}_{m,j}^2 + \left(\frac{\hat{\varphi}_{m,j+1}-\hat{\varphi}_{m,j-1}}{2}\right)^2 - \frac{\hat{\varphi}_{m,j}}{2j}\left(\hat{\varphi}_{m,j+1}-\hat{\varphi}_{m,j-1}\right) + (1+4m^2)\frac{\hat{\varphi}_{m,j}^2}{j^2}\right.$$

*email: santhu@iisertvm.ac.in
†email: shanki@phy.iitb.ac.in

$$
\begin{aligned}
&+\frac{\epsilon}{P}\left\{\left(\hat{\varphi}_{m,j+1}-2\hat{\varphi}_{m,j}+\hat{\varphi}_{m,j-1}\right)^2+\frac{2\beta}{j^2}\left(\hat{\varphi}_{m,j+1}-2\hat{\varphi}_{m,j}+\hat{\varphi}_{m,j-1}\right)^2+\frac{\beta^2}{j^4}\hat{\varphi}_{m,j}^2\right\} \\
&+\frac{\tau}{P^2}\left\{\left(\frac{\hat{\varphi}_{m,j+2}-2\hat{\varphi}_{m,j+1}+2\hat{\varphi}_{m,j-1}-\hat{\varphi}_{m,j-2}}{2}\right)^2+\frac{\alpha}{j^2}\left(\hat{\varphi}_{m,j+1}-2\hat{\varphi}_{m,j}+\hat{\varphi}_{m,j-1}\right)^2\right. \\
&+\frac{\beta^2}{j^4}\left(\frac{\hat{\varphi}_{m,j+1}-\hat{\varphi}_{m,j-1}}{2}\right)^2+\frac{25/4+m^2}{j^6}\beta^2\hat{\varphi}_{m,j}^2-\frac{5\beta^2}{2j^5}\left(\frac{\hat{\varphi}_{m,j+1}-\hat{\varphi}_{m,j-1}}{2}\right)\hat{\varphi}_{m,j} \\
&-\left(\frac{\hat{\varphi}_{m,j+2}-2\hat{\varphi}_{m,j+1}+2\hat{\varphi}_{m,j-1}-\hat{\varphi}_{m,j-2}}{2j}\right)\left(\hat{\varphi}_{m,j+1}-2\hat{\varphi}_{m,j}+\hat{\varphi}_{m,j-1}\right) \\
&+\frac{2\beta}{j^2}\left(\frac{\hat{\varphi}_{m,j+2}-2\hat{\varphi}_{m,j+1}+2\hat{\varphi}_{m,j-1}-\hat{\varphi}_{m,j-2}}{2}\right)\left(\frac{\hat{\varphi}_{m,j+1}-\hat{\varphi}_{m,j-1}}{2}\right) \\
&-\frac{5\beta}{j^3}\left(\frac{\hat{\varphi}_{m,j+2}-2\hat{\varphi}_{m,j+1}+2\hat{\varphi}_{m,j-1}-\hat{\varphi}_{m,j-2}}{2}\right)\hat{\varphi}_{m,j}-\frac{\beta}{j^3}\left(\hat{\varphi}_{m,j+1}-2\hat{\varphi}_{m,j}+\hat{\varphi}_{m,j-1}\right) \\
&\left.\left.\times\left(\frac{\hat{\varphi}_{m,j+1}-\hat{\varphi}_{m,j-1}}{2}\right)+\frac{\left(5/2+2m^2\right)\beta}{j^4}\left(\hat{\varphi}_{m,j+1}-2\hat{\varphi}_{m,j}+\hat{\varphi}_{m,j-1}\right)\hat{\varphi}_{m,j}\right\}\right]
\end{aligned} \tag{5}
$$

where $\pi_{m,j} = a\,\Pi_{m,j}$ is the new canonical momentum field, and $\hat{\varphi}_{m,N+1} = 0$ is the constraint on field.

The canonical commutation relation between the dimensionless fields is

$$[\hat{\varphi}_{m,j}, \hat{\pi}_{m',j'}] = i\delta_{mm'}\delta_{jj'} \tag{6}$$

More explicitly we can write the above interacting quantum Hamiltonian as a set of N coupled harmonic oscillators with time independent frequency as [2, 3],

$$H(P) = \frac{1}{2a}\sum_{m=-\infty}^{\infty}\sum_{i,j=1}^{N}\left[\pi_{m,i}^2\,\delta_{i,j} + \phi_{m,i}\,K_{ij}(P,m)\,\phi_{m,j}\right] \tag{7}$$

where K_{ij} is a real symmetric interaction matrix have positive real energy eigenvalues and is given by,

$$
\begin{aligned}
K_{ij}(P,m) \;=\;& K^{(1)}\delta_{1,j}+K^{(2)}\delta_{2,j}+K^{(3)}\delta_{i,j(j\neq 1,2,N-1,N)}+K^{(4)}\delta_{N-1,j}+K^{(5)}\delta_{N,j} \\
& K^{(6)}\left(\delta_{1,j-1}+\delta_{2,j+1}\right)+K^{(7)}\left(\delta_{i,j-1(j\neq 2)}+\delta_{i,j+1(i\neq 2)}\right)+K^{(8)}\left(\delta_{i+1,N}+\delta_{N,j+1}\right) \\
& +K^{(9)}\left(\delta_{i+2,j}+\delta_{i,j+2}\right)+K^{(10)}\left(\delta_{i+3,j}+\delta_{i,j+3}\right)+K^{(11)}\left(\delta_{i+4,j}+\delta_{i,j+4}\right)
\end{aligned} \tag{8}
$$

where

$$K^{(1)} = \frac{1}{4}+\frac{\alpha}{j^2}+\epsilon P\left(5-\frac{4\beta}{j^2}+\frac{\beta^2}{j^4}\right)+\tau P^2\left(\frac{3}{4}+\frac{17\alpha}{(j+1)^2}+\frac{\gamma\beta^2}{j^6}-\frac{2\varsigma\beta}{j^4}-\frac{3\beta}{2(j+1)^3}+\frac{\beta^2}{4(j+1)^4}\right) \tag{9a}$$

$$K^{(2)} = \frac{1}{2}+\frac{\alpha}{j^2}+\epsilon P\left(6-\frac{4\beta}{j^2}+\frac{\beta^2}{j^4}\right)+\tau P^2\left(\frac{35}{12}+\frac{19\alpha}{(j+1)^2}+\frac{\gamma\beta^2}{j^6}-\frac{2\varsigma\beta}{j^4}-\frac{43\beta}{(j+1)^3}+\frac{82\beta^2}{4(j+1)^4}\right) \tag{9b}$$

$$
\begin{aligned}
K^{(3)} =\;& \frac{1}{2}+\frac{\alpha}{j^2}+\epsilon P\left(6-\frac{4\beta}{j^2}+\frac{\beta^2}{j^4}\right)+\tau P^2\left(\frac{5}{2}+\frac{2}{j^2-1}+\frac{4\alpha}{j^2}+\frac{\gamma\beta^2}{j^6}-\frac{2\varsigma\beta}{j^4}+(\alpha-\beta)\right. \\
& \left.\times\left(\frac{1}{(j+1)^2}+\frac{1}{(j-1)^2}\right)+\frac{\beta^2}{4}\left(\frac{2}{(j+1)^4}+\frac{1}{(j-1)^4}\right)+\frac{\beta}{2}\left(\frac{1}{(j+1)^3}-\frac{1}{(j-1)^3}\right)\right)
\end{aligned} \tag{9c}
$$

$$
\begin{aligned}
K^{(4)} =\;& \frac{1}{2}+\frac{\alpha}{(N-1)^2}+\epsilon P\left(6-\frac{4\beta}{(N-1)^2}+\frac{\beta^2}{(N-1)^4}\right)+\tau P^2\left(\frac{9}{4}+\frac{2}{(N-2)N}+(\alpha-\beta)\right. \\
& \times\left(\frac{1}{(N-2)^2}+\frac{1}{N^2}\right)+\frac{\beta^2}{4}\left(\frac{1}{(N-2)^4}+\frac{2}{N^4}\right)+\frac{4\alpha}{(N-1)^2}+\frac{\gamma\beta^2}{(N-1)^6}-\frac{2\varsigma\beta}{(N-1)^4} \\
& \left.+\frac{\beta}{2}\left(\frac{1}{N^3}-\frac{1}{(N-1)^3}\right)\right)
\end{aligned} \tag{9d}
$$

$$K^{(5)} = \left(\frac{1}{4}+\frac{\alpha}{N^2}\right)+\epsilon P\left(5-\frac{4\beta}{N^2}+\frac{\beta^2}{N^4}\right)+\tau P^2\left(\frac{5N+1}{4(N-2)}+\frac{\alpha-\beta}{(N-1)^2}+\frac{\beta^2}{4(N-1)^4}-\frac{\beta}{2(N-1)^3}\right.$$

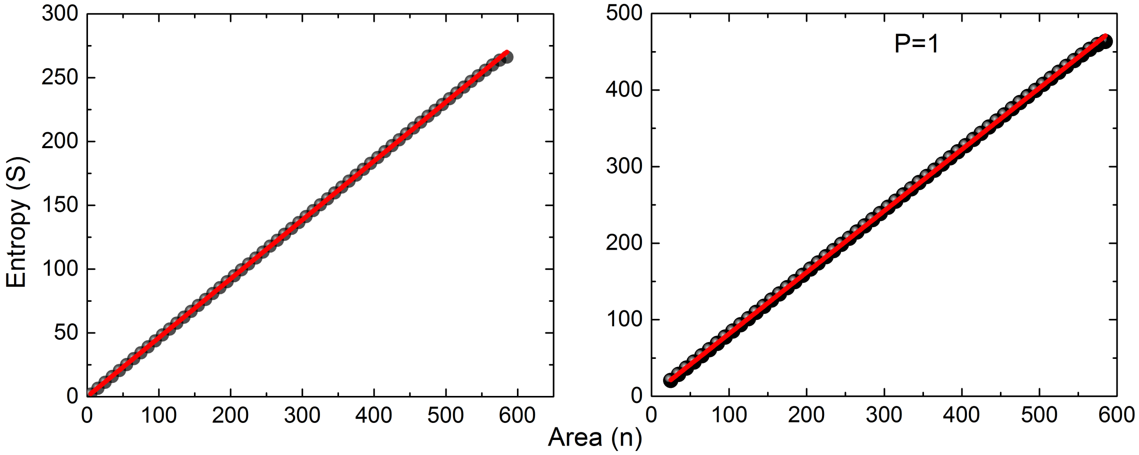

Fig. 1: Left figure is the plot of entanglement entropy (S) vs n for linear dispersion relation, $\omega = k$. Right figure is the plot of entanglement entropy (S) vs n for $\omega^2 = k^2 + k^4/\kappa^2$. In all cases we set $N = 600$. The black dots represent numerical data points and the red line is the best linear fit represented by the equation $S = a\,n + 0.038\,\log n$, where a is slope of the line for both the cases. This is consistent with the results of [4]. The expression for the entropy, the fitted red line, is a universal property of entanglement entropy for $z = 2$ class systems.

$$
\begin{aligned}
& + \frac{\gamma\beta^2}{N^6} - \frac{2\beta\varsigma}{N^4}\Bigg) && \text{(9e)}\\
K^{(6)} &= -\frac{1}{8} + \epsilon P\left(\frac{5\beta}{4} - 4\right) + \frac{\tau P^2}{2}\left(\frac{-11}{6} - 5\alpha + \frac{191\beta}{36} + \frac{17\beta\varsigma}{16} - \frac{155\beta^2}{64}\right) && \text{(9f)}\\
K^{(7)} &= -\frac{1}{4i(i+1)} + \epsilon P\left(-4 + \beta\left(\frac{1}{i^2} + \frac{1}{(i+1)^2}\right)\right) + \frac{\tau P^2}{2}\Bigg(-2 - \frac{3}{(i-1)(i+2)} \\
& - \frac{1}{i(i+1)} + \frac{\beta}{2(i-1)^2} - \frac{4\alpha}{i^2} + \frac{\varsigma\beta}{i^4} + \frac{6\beta}{i^3} - \frac{5\beta^2}{2i^5} - \frac{4\alpha}{(i+1)^2} + \frac{\varsigma\beta}{(i+1)^4} + \frac{6\beta}{(i+1)^3} \\
& - \frac{5\beta^2}{2(i+1)^5} - \frac{4\alpha}{(i+1)^2} + \frac{\beta}{2(i+1)^2}\Bigg) && \text{(9g)}\\
K^{(8)} &= K^{(7)} + \left(1 - \frac{1}{2(N+1)} - \frac{\beta}{2(N+1)^2}\right) && \text{(9h)}\\
K^{(9)} &= -\frac{1}{4} + \epsilon P + \frac{\tau P^2}{2}\left(-2 + \frac{2\alpha}{(i+1)^2} - \frac{\beta^2}{2(i+1)^4} + \frac{2}{i(i+2)} + \frac{5\beta}{2}\left(\frac{1}{(i+2)^3} - \frac{1}{i^3} + \frac{4\beta}{5(i+1)^2}\right)\right) && \text{(9i)}\\
K^{(10)} &= \frac{\tau P^2}{2}\left[2 - \frac{1}{(i+1)(i+2)} - \frac{\beta}{2}\left(\frac{1}{(i+1)^2} + \frac{1}{(i+2)^2}\right)\right] && \text{(9j)}\\
K^{(11)} &= -\frac{\tau P^2}{4} && \text{(9k)}
\end{aligned}
$$

and

$$
\alpha = \frac{1+4m^2}{4}, \quad \beta = \frac{1-4m^2}{4}, \quad \gamma = \frac{25}{4} + m^2, \quad \varsigma = \frac{5}{2} + 2m^2 \qquad \text{(10a)}
$$

It is important to note that the interaction matrix K_{ij} contains all the diagonal entries $(K^{(1)}, K^{(2)}, K^{(3)}, K^{(4)}, K^{(5)})$, nearest neighbour couplings $(K^{(6)}, K^{(7)}, K^{(8)})$, NN couplings $(K^{(9)})$ and NNN couplings $(K^{(10)}, K^{(11)})$. The NNN couplings are from the sixth order derivative term.

We evaluate the entropy using the method discussed in Refs. [2, 3] using the von-Neumann expression as $S = -\mathrm{Tr}\,\rho\log\rho$. In all numerical computations are done in MATLAB R2012a, we use 10^{-8} as a numerical accuracy and all matrix entries in the K_{ij} are rescaled by a factor of 10^{-10}. Figure (1) contains the plot of EE versus n for the linear dispersion relation $(\omega = k)$ (left plot) and $\omega^2 = k^2 + k^4/\kappa^2$ (right plot).

It is interesting to note that EE is proportional to area of the boundary, that is n for the case of nearest neighbour and NN coupling, however, its profile completely changes in the case of NNN coupling.

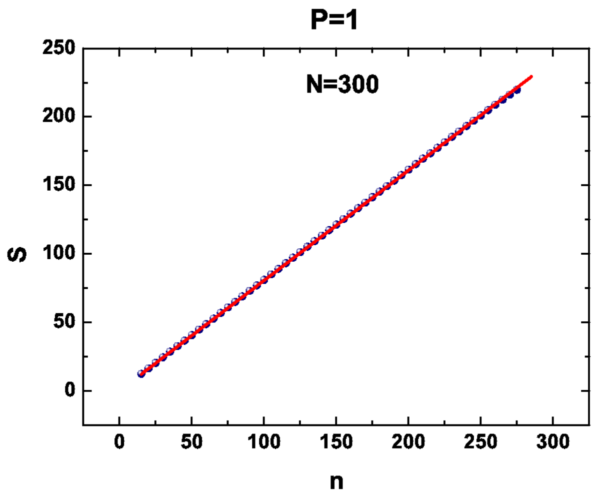

(a)

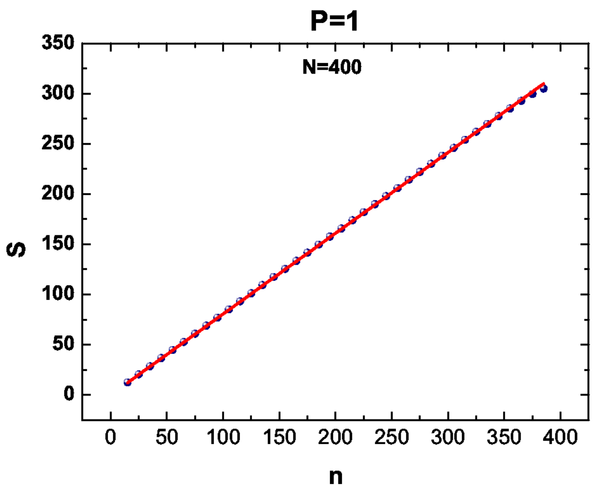

(b)

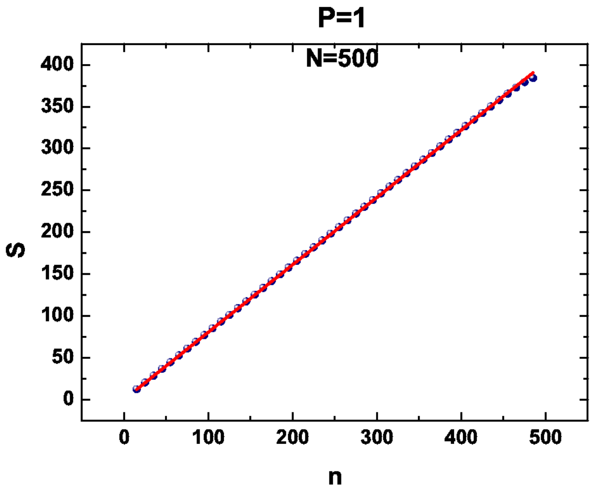

(c)

Fig. 2: Plot of entropy as a function of n for various P values for $\epsilon = 1, \tau = 0$ with N=300,400, and 500 sites respectively arranged in sub figures (a), (b), and (c). The blue dots in each plot represents numerical data and the red line is the best linear fit represented by $S = a\,n + 0.038\,\log n$, where a is slope of the line. These plots are consistent with the analysis reported in Ref. [4]

II. ENTANGLEMENT ENTROPY FOR DIFFERENT LATTICE SIZES

The following figures represent the behavior of EE for different N's. Plots in Fig. (2) are for the dispersion relation $\omega^2 = k^2 + k^4/\kappa^2$ and the plots in Fig. (3) are for the dispersion relation $\omega^2 = k^2 + k^4/\kappa^2 + k^6/\kappa^4$. It is clear for the former case that the entropy scales with area and the next leading order is the diverging logarithmic correction which was reported for $z = 2$ critical theories in two dimensions [4]. However, in case (II), the we observe a violation in area-law due to the NNN couplings and it is shown in Fig. (3) for various N values.

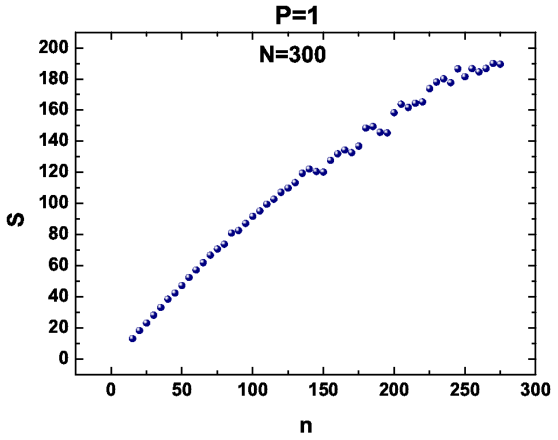

(a)

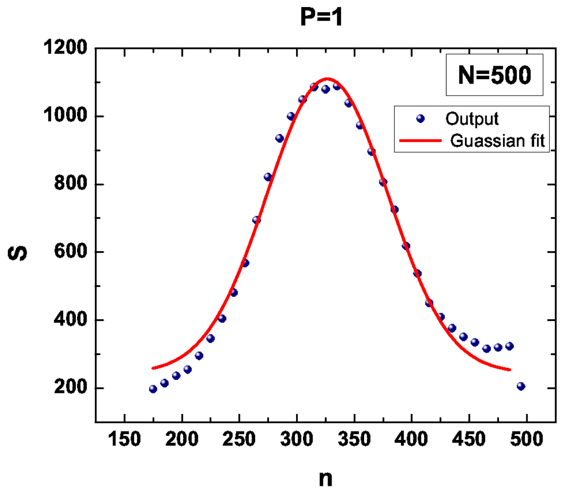

(b)

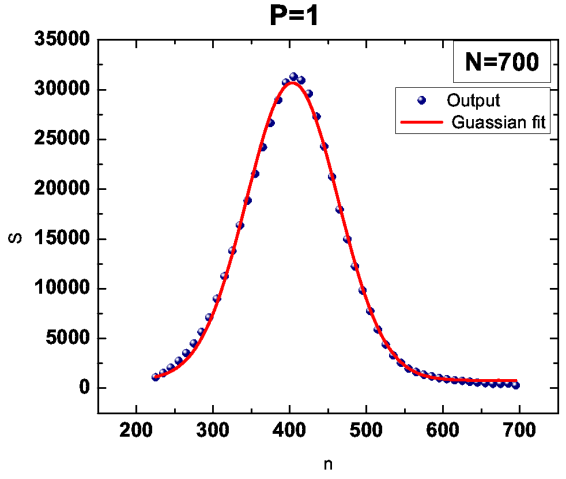

(c)

Fig. 3: Plot of entropy as a function of n for various P values for $\epsilon = 1, \tau = 1$ with $N = 300, 500,$ and 700 sites respectively arranged in sub figures (a), (b), and (c). The blue dots represent the output and red colour is the best fit in figs. (b) and (c).

III. ENTANGLEMENT ENTROPY OF A CYLINDER

The Hamiltonian in the 3-dimensional cylindrical geometry is given by:

$$\hat{H}_1 = \frac{1}{2}\int d^2\mathbf{r}\, dz \left[\hat{\Pi}^2(\mathbf{r}, z) + \left|\nabla\hat{\Phi}(\mathbf{r}, z)\right|^2 + \frac{\epsilon}{\kappa^2}\left|\nabla^2_{\mathbf{r}}\hat{\Phi}(\mathbf{r}, z)\right|^2 + \frac{\tau}{\kappa^4}\left|\nabla^3_{\mathbf{r}}\hat{\Phi}(\mathbf{r}, z)\right|^2\right] \tag{11}$$

where $\hat{\Phi}$ and $\hat{\Pi}$ are the one component real massless scalar field and its conjugate momenta respectively, ϵ and τ are dimensionless constants, κ has the dimension of wave number and $\mathbf{r}(= r, \theta)$ is the circular polar coordinates and z is the height of the cylinder. Here $\nabla^2_{\mathbf{r}}$ and $\nabla^3_{\mathbf{r}}$ represent higher order derivative operators for the 2-D plane. That is we study the effect of the higher derivative terms in the 2-D plane —- interaction strength is comparatively less in the z direction.

The equal time canonical commutation relation between fields, which are the functions of $\mathbf{r}$ and z is given by,

$$\left[\hat{\Phi}(\mathbf{r}, z), \hat{\Pi}(\mathbf{r}', z)\right] = i\,\delta^2(\mathbf{r} - \mathbf{r}')\,\delta(z - z') = \frac{i}{r}\delta(r - r')\,\delta(\theta - \theta')\,\delta(z - z') \tag{12}$$

We use the following ansatz for expanding the real scalar fields in circular cylindrical coordinates

$$\hat{\Pi}(\mathbf{r}, z) = \frac{1}{2\pi^2} \int_0^\infty dk \sum_{m=-\infty}^{\infty} \frac{\hat{\Pi}_m(r,k)}{\sqrt{r}} \cos m\theta \ \cos kz \tag{13a}$$

$$\hat{\Phi}(\mathbf{r}, z) = \frac{1}{2\pi^2} \int_0^\infty dk \sum_{m=-\infty}^{\infty} \frac{\hat{\varphi}_m(r,k)}{\sqrt{r}} \cos m\theta \ \cos kz, \tag{13b}$$

where m is the angular momentum quantum number and k is a real positive parameter which has the dimension of momentum. The canonical commutation relation between the new rescaled fields is

$$\left[\hat{\varphi}_m(r,k), \hat{\Pi}_{m'}(r',k')\right] = i\,\delta(r-r')\,\delta(k-k')\,\delta_{mm'} \tag{14}$$

Unlike in the case of circular polar coordinates, here we have to do two different discretization —one for the radial direction and other along in the height of the cylinder. For numerical simplicity, we choose the height of the cylinder is some arbitrary number ν times its radius. Let a and b are the lattice parameter in the radial and z- direction respectively and here we takes $b = \nu\ a$. That is,

$$r = ja; \qquad 1/k = s\ \nu\ a \tag{15}$$

After some little algebra, we can write the total Hamiltonian in the following form;

$$\begin{aligned}
\hat{H}_1 \ = \ & \frac{1}{2a} \sum_{s=1}^{N_1} \sum_{m=-\infty}^{\infty} \sum_{j=1}^{N} \Bigg[\hat{\Xi}^2_{m,j,s} + (1+4m^2)\frac{\hat{\chi}^2_{m,j,s}}{j^2} + \frac{1}{s^2\nu^2}\hat{\chi}^2_{m,j,s} \\
& + \left(\frac{\hat{\chi}_{m,j+1,s} - \hat{\chi}_{m,j-1,s}}{2}\right)^2 - \frac{\hat{\chi}_{m,j,s}}{2j}\left(\hat{\chi}_{m,j+1,s} - \hat{\chi}_{m,j-1,s}\right) \\
& + \frac{\epsilon}{P}\left\{ \left(\hat{\chi}_{m,j+1,s} - 2\hat{\chi}_{m,j,s} + \hat{\chi}_{m,j-1,s}\right)^2 + \frac{2\beta}{j^2}\left(\hat{\chi}_{m,j+1,s} - 2\hat{\chi}_{m,j,s} + \hat{\chi}_{m,j-1,s}\right)^2 + \frac{\beta^2}{j^4}\hat{\chi}^2_{m,j,s} \right\} \\
& + \frac{\tau}{P^2}\Bigg\{ \left(\frac{\hat{\chi}_{m,j+2,s} - 2\hat{\chi}_{m,j+1,s} + 2\hat{\chi}_{m,j-,s1} - \hat{\chi}_{m,j-2,s}}{2}\right)^2 + \frac{\alpha}{j^2}\left(\hat{\chi}_{m,j+1,s} - 2\hat{\chi}_{m,j,s} + \hat{\chi}_{m,j-1,s}\right)^2 \\
& + \frac{\beta^2}{j^4}\left(\frac{\hat{\chi}_{m,j+1,s} - \hat{\chi}_{m,j-1,s}}{2}\right)^2 + \frac{25/4 + m^2}{j^6}\beta^2\hat{\chi}^2_{m,j,s} - \frac{5\beta^2}{2j^5}\left(\frac{\hat{\chi}_{m,j+1,s} - \hat{\chi}_{m,j-1,s}}{2}\right)\hat{\chi}_{m,j,s} \\
& - \left(\frac{\hat{\chi}_{m,j+2,s} - 2\hat{\chi}_{m,j+1,s} + 2\hat{\chi}_{m,j-1,s} - \hat{\chi}_{m,j-2,s}}{2j}\right)\left(\hat{\chi}_{m,j+1,s} - 2\hat{\chi}_{m,j,s} + \hat{\chi}_{m,j-1,s}\right) \\
& + \frac{2\beta}{j^2}\left(\frac{\hat{\chi}_{m,j+2,s} - 2\hat{\chi}_{m,j+1,s} + 2\hat{\chi}_{m,j-1,s} - \hat{\chi}_{m,j-2,s}}{2}\right)\left(\frac{\hat{\chi}_{m,j+1,s} - \hat{\chi}_{m,j-1,s}}{2}\right) \\
& - \frac{5\beta}{j^3}\left(\frac{\hat{\chi}_{m,j+2,s} - 2\hat{\chi}_{m,j+1,s} + 2\hat{\chi}_{m,j-1,s} - \hat{\chi}_{m,j-2,s}}{2}\right)\hat{\chi}_{m,j,s} - \frac{\beta}{j^3}\left(\hat{\chi}_{m,j+1,s} - 2\hat{\chi}_{m,j,s} + \hat{\chi}_{m,j-1,s}\right) \\
& \times \left(\frac{\hat{\chi}_{m,j+1,s} - \hat{\chi}_{m,j-1,s}}{2}\right) + \frac{\left(5/2 + 2m^2\right)\beta}{j^4}\left(\hat{\chi}_{m,j+1,s} - 2\hat{\chi}_{m,j,s} + \hat{\chi}_{m,j-1,s}\right)\hat{\chi}_{m,j,s} \Bigg\}\Bigg]
\end{aligned} \tag{16}$$

where $\Xi_{m,j} = \sqrt{a}\ \Pi_{m,j}$ is the new canonical momentum field, $\chi_{m,j} = \varphi_{m,j,p}/\sqrt{a}$ is the new dimensionless scalar field, N_1 and N are the lattice points along the radial, z directions respectively and $\chi_{m,N_1+1} = 0, \chi_{m,N+1} = 0$, are the constraints on field. Thus the above Hamiltonian can be bring into a form of system of coupled HO's;

$$H_1(P) = \frac{1}{2a} \sum_{s=1}^{\infty} \sum_{m=-\infty}^{N_1} \sum_{i,j=1}^{N} \left[\Xi^2_{m,s,i}\ \delta_{i,j} + \chi_{m,s,i}\ T_{ij}(P,m,s)\ \chi_{m,s,j}\right] \tag{17}$$

where $T_{ij}(P,m,s) = K_{ij}(P,m) + (s\ \nu)^{-2}\,\delta_{i,j}$ is a real symmetric interaction matrix have positive real energy eigenvalues. Using the same procedure, we obtain ground state entanglement entropy. Fig. 4 contains the plot of entanglement entropy versus area for scenario $\omega^2 = k^2 + k^4/\kappa^2$ and $\omega^2 = k^2 + k^4/\kappa^2 + k^6/\kappa^4$ respectively, where k is the three dimensional wave vector.

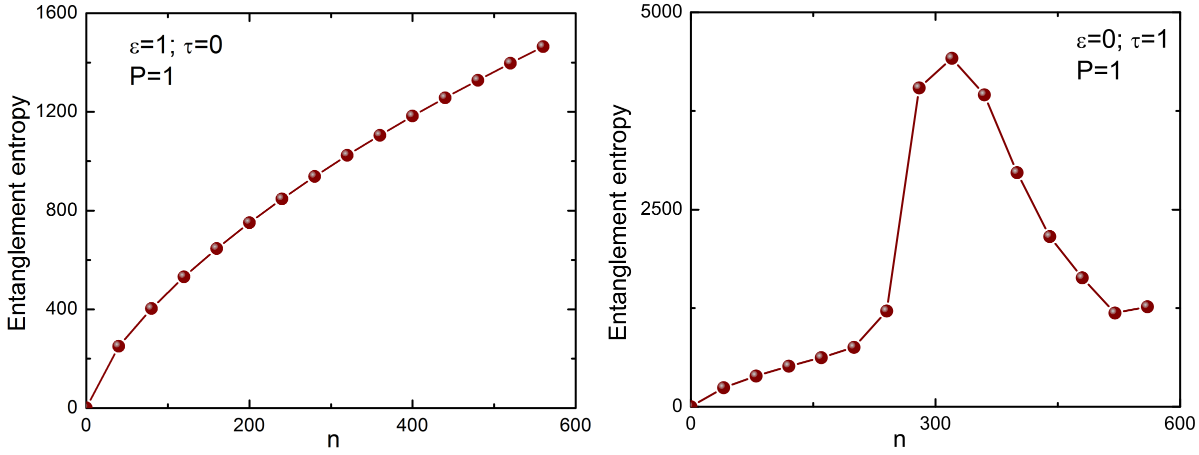

(a)

(b)

Fig. 4: Left figure is the plot of entanglement entropy versus area for scenario I. Right figure is the plot of entanglement entropy versus area for scenario II. We have used the lattice size in the z-axis is 10 times the lattice size in the 2-D plane. The number of lattice points used in the calculation are $N = 600, N_1 = 2, \delta P = 10^{-9}$ and the accuracy is 10^{-2}.

IV. SPECIFIC HEAT AND FIDELITY SUSCEPTIBILITY FROM ENTANGLEMENT ENTROPY

Rényi entropy is given by

$$S_\varsigma = \frac{1}{1-\varsigma} \log \mathrm{Tr}\, \rho^\varsigma \tag{18}$$

where ς is the Rényi parameter and $\varsigma \to 1$ leads to von-Neumann entanglement entropy. $S_{\varsigma\to\infty}$ is referred in the literature as single copy entanglement entropy [5] and quantifies quantum correlations in the quantum ground state of the many body systems. The single copy entanglement entropy is given by,

$$S_{m,s,\varsigma\to\infty} = -\sum_{i=1}^{N-n} \log\left(1-\xi_i\right) \tag{19}$$

where ξ_i has the same structure as that in the circular case and is obtained from $T_{ij}(P, m, s)$.

It has been interpreted that there has been a close resemblance between the single copy entanglement entropy and the thermodynamic entropy [6, 7]. One can define entanglement specific heat as,

$$C_{ent} = P\frac{dS_{\varsigma\to\infty}}{dP} \tag{20}$$

Fidelity susceptibility is defined as

$$\chi_F = \lim_{\delta P\to 0}\left[-2\,\frac{\delta^2 \log F}{\delta P^2}\right] \tag{21}$$

where $F = \langle\psi_{1\mathrm{GS}}(P+\delta P)|\psi_{1\mathrm{GS}}(P)\rangle$ is the fidelity of the system and $\psi_{1\mathrm{GS}}$ is the ground state wave function of the Hamiltonian H_1.

A. Implications for high temperature superconductivity

It is natural to ask: Is there any physical system that has a long-range interaction in the 2-D plane and shows such a distinct phase transition? One system is Copper Oxide HTS which has 2-D layered crystal structure whose inter-atomic distance is smaller than the inter-atomic distance along the z-axis [8]. It is long-known that, in HTS, the coloumbic interactions between the electrons tend to make an anti-ferromagnetic arrangement of spins in the Copper Oxide planes and the magnetic transition is controlled by the weak coupling between the planes along the z-axis [9].

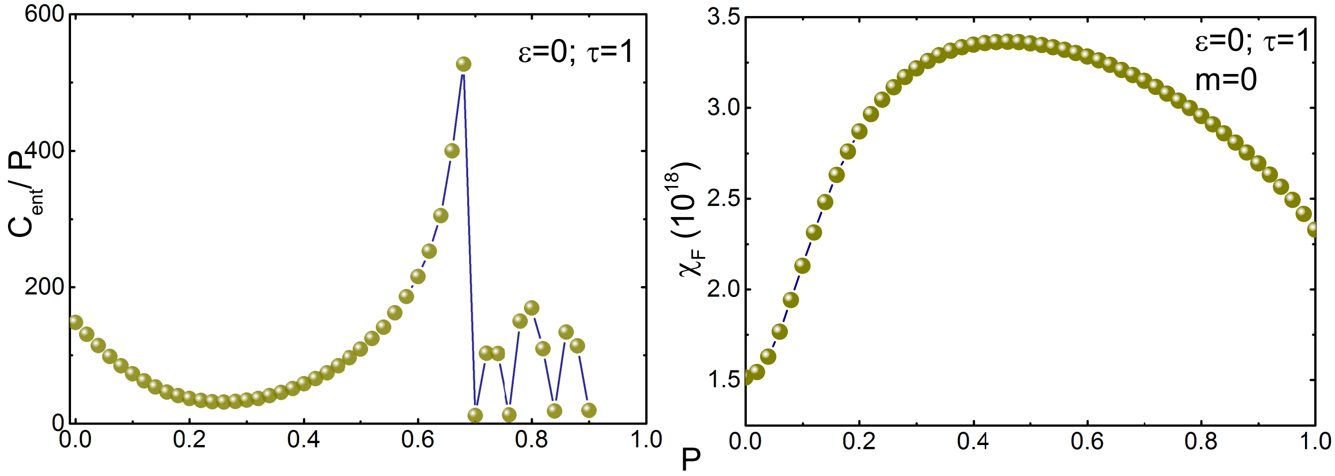

Fig. 5: Left figure is the plot of entanglement specific heat per P ($C_{ent}/P = dS_{\varsigma\to\infty}/dP$) versus P obtained from the single copy entanglement entropy (S_∞ is the Rényi entropy having infinity as the Rényi index). Right figure is the plot of fidelity susceptibility ($\chi_F = \lim_{\delta P\to 0} -2\log \delta^2 F/\delta P^2$) as a function of P. We have taken lattice size of the z-axis to be 10 times more than the lattice size of the 2-dimensional surface, $N = 600, n = 300$ and $\delta P = 10^{-7}$.

To overcome the complexity of the interactions, we consider a scalar order parameter Φ in 3-dimensional cylindrical geometry such that the higher derivative terms contribute only in the 2-dimensional plane while the first derivative term contribute acts in all the three spatial dimensions. Repeating the analysis in 3-dimensions, it can be shown that the model in 3-dimensions has the same entropy profile as that in the 2-dimensional case. In Fig. (5), we have plotted entanglement specific heat and fidelity susceptibility defined in Eqs. (20) and (21) as a function of P.

Following points are interesting to note: First, the entanglement specific heat shows discontinuity at a particular value of P. This is indeed similar to the discontinuity of the specific heat measurement of the single crystals of $YBa_2Cu_3O_{7-\delta}$ [8]. Second, discontinuity of the entanglement specific heat at, say, P_0 implies that the correlation length diverges close to P_0. For instance, in the case of transverse quantum Ising model, entanglement specific heat diverges logarithmically [10] that signals the correlation length to diverge. In our case, the entanglement specific heat diverges more like power-law. Third, it is interesting to see how our model fares with the specific heat measurements at extreme high magnetic fields as recently reported by Badoux et al [11].

---

[1] S. Ghosh and S. Shankaranarayanan, "Entanglement signatures of phase transition in higher-derivative quantum field theories",*Phys. Rev. D* **86** (Dec, 2012) 125011.

[2] L. Bombelli, R. K. Koul, J. Lee, and R. D. Sorkin, "Quantum source of entropy for black holes",*Phys. Rev. D* **34** (Jul, 1986) 373–383.

[3] M. Srednicki, "Entropy and area",*Phys. Rev. Lett.* **71** (Aug, 1993) 666–669.

[4] E. Fradkin and J. E. Moore, "Entanglement entropy of 2d conformal quantum critical points: Hearing the shape of a quantum drum",*Phys. Rev. Lett.* **97** (Aug, 2006) 050404.

[5] J. Eisert and M. Cramer, "Single-copy entanglement in critical quantum spin chains",*Phys. Rev. A* **72** (Oct, 2005) 042112.

[6] K. K. R. Seroje, R. S. dela Rosa, and F. N. C. Paraan, "Effective thermodynamics of isolated entangled squeezed and coherent states", *European Journal of Physics* **36** (2015) no. 5, 055051.

[7] J.-M. Stphan, G. Misguich, and V. Pasquier, "Rnyi entanglement entropies in quantum dimer models: from criticality to topological order", *Journal of Statistical Mechanics: Theory and Experiment* **2012** (2012) no. 02, P02003.

[8] N. Overend, M. A. Howson, and I. D. Lawrie, "3d X-Y scaling of the specific heat of yba$_2$ cu$_3$ o$_{7-\delta}$ single crystals",*Phys. Rev. Lett.* **72** (May, 1994) 3238–3241.

[9] D. A. Bonn, "Are high-temperature superconductors exotic?",*Nat Phys* **2** (March, 2006) 159–168.

[10] A. Osterloh, L. Amico, G. Falci, and R. Fazio, "Scaling of entanglement close to a quantum phase transition",*Nature* **416** (Dec, 2002) 608.

[11] Badoux S., Tabis W., Lalibert F., Grissonnanche G., Vignolle B., Vignolles D., Bard J., Bonn D. A., Hardy W. N., Liang R., Doiron-Leyraud N., Taillefer Louis, and Proust Cyril*Nature* **531** (Mar., 2016) 210–214.
